# Walking Performance: Correlation between Energy Cost of Walking and Walking Participation. New Statistical Approach Concerning Outcome Measurement

**DOI:** 10.1371/journal.pone.0056669

**Published:** 2013-02-28

**Authors:** Marco Franceschini, Anais Rampello, Maurizio Agosti, Maurizio Massucci, Federica Bovolenta, Patrizio Sale

**Affiliations:** 1 Department of NeuroRehabilitation IRCCS San Raffale, Pisana, Rome; 2 Department of Rehabilitation, University Hospital of Parma, Parma, Italy; 3 Rehabilitation Unit, Hospital of Passignano, Passignano, Perugia, Italy; 4 Medicine Rehabilitation NOCSAE Hospital AUSL of Modena, Modena, Italy; Hospital Nacional de Parapléjicos, Spain

## Abstract

Walking ability, though important for quality of life and participation in social and economic activities, can be adversely affected by neurological disorders, such as Spinal Cord Injury, Stroke, Multiple Sclerosis or Traumatic Brain Injury. The aim of this study is to evaluate if the energy cost of walking (CW), in a mixed group of chronic patients with neurological diseases almost 6 months after discharge from rehabilitation wards, can predict the walking performance and any walking restriction on community activities, as indicated by Walking Handicap Scale categories (WHS). One hundred and seven subjects were included in the study, 31 suffering from Stroke, 26 from Spinal Cord Injury and 50 from Multiple Sclerosis. The multivariable binary logistical regression analysis has produced a statistical model with good characteristics of fit and good predictability. This model generated a cut-off value of.40, which enabled us to classify correctly the cases with a percentage of 85.0%. Our research reveal that, in our subjects, CW is the only predictor of the walking performance of in the community, to be compared with the score of WHS. We have been also identifying a cut-off value of CW cost, which makes a distinction between those who can walk in the community and those who cannot do it. In particular, these values could be used to predict the ability to walk in the community when discharged from the rehabilitation units, and to adjust the rehabilitative treatment to improve the performance.

## Introduction

Walking ability can be adversely affected by neurological disorders, such as Spinal Cord Injury (SCI), Stroke, Multiple Sclerosis (MS) or Traumatic Brain Injury (TBI) [1]. Walking recovery is one of the most important goals of rehabilitation treatment for neurological and/or orthopaedic diseases [2]–[5]. From the perspective of the patients, walking is not more relevant than the ability to walk in the community independently [6], [7] but several factors interfere with walking recovery from neurological diseases. The main one is the high energy cost of gait due to muscular weakness and consequent biomechanical inefficiency [8]. The complexity of environmental factors is another aspect [9] that makes it difficult to use skills hard earned in rehabilitation setting. The roles of the environmental factors, such as barriers of facilitators, were emphasized by the International Classification of Functioning and Disability and Health [10]. This framework distinguishes the “capacity” as the theoretical ability of walking if the environment were uniform or standard (environment without barriers or facilitators) from the “performance” that relates to what a person does in the environmental context in which he actually lives. One of the most important objectives of rehabilitation is to reduce the gap between walking capacity and walking performance. Various clinical scales were carried out to asses and to predict the walking abilities of people suffering from neurological disease. On the basis of the gait speed and the self-reported ability to walk in the community of a group of post-stroke people, Perry and colleagues [11] have created a Walking Handicap Scale (WHS), a classification of 6 functional walking categories, 3 of which refer to community ambulation. In particular, the WHS was performed to offer quantitative method of relating the social disadvantage of patients to the impairment and disability sustained [11]. It could be important to provide community-walking performance of the patient on the basis of walking capacity acquired in the rehabilitation unit. Concerning the biomechanical aspects of walking, many works have considered that the gait velocity, the activity monitors and the spatiotemporal parameters can predict different skills of community ambulation [12]–[13]. Shumway-Cook and colleagues emphasize the importance of temporal factors, postural transitions, external physical load and terrain [12]. However, the energy cost of walking represents a good indicator of overall exercise performance of walking, which should be considered when evaluating a patient’s functional independence [14].

The aim of this study is to evaluate if the energy cost of walking (CW) in a mixed group of patients with neurological diseases, almost 6 months after discharge from rehabilitation wards, can predict the walking performance and the walking restriction to participate in the community, as indicated by Walking Handicap Scale categories (WHS) [11].

## Methods

### Design

Cross-sectional study.

### Sample

From January 2007 to December 2009 we recruited outpatient subjects with Stroke, Multiple Sclerosis and Spinal Cord Injury, all with walking limitations. The inclusion criteria were: (a) age >18 years at the beginning of the study; (b) almost 6 months after conclusion of a programmed rehabilitation plan; (c) return home after discharge; (d) ability to walk independently, with residual difficulty for at least 6 minutes, with or without walking aids. The exclusion criteria were: (a) presence of cardio respiratory co-morbidity; (b) presence of orthopaedic co-morbidity (c) patients who refused the consent to take part in the study.

### Sample Size

Supported by literature, we calculated the power of the sample based on 15 subjects for each independent variable (predictor) used in the regression [15]. In particular, the following independent variables (Age, Sex, Etiology [Stroke, MS, SCI] and energy cost) were inserted in the regression model (binary logistic). According to these parameters, which therefore considered 6 independent variables for 15 subjects, at least 90 cases were required and we analysed 107 subjects.

### Main Outcome Measures

Walking Handicap Scale (WHS) and the energy cost of walking (CW).

### Testing Protocol

The local Ethics Committee approved the study. All clinical assessments and tests were performed in rehabilitation hospital and all patients gave written informed consent. A blinded examiner assessed clinical and metabolic evaluation of walking at the moment of the inclusion. The severity of MS was evaluated in accordance with EDSS scale, while for the SCI we referred to the Asia impairment scale. The Stroke group were classified in mild, moderate and severe according to FIM Score (mild >80, moderate 40–80 and severe <40). The clinical evaluation of gait ability was performed according to Walking Handicap Scale (WHS) [16], which was then dichotomized into two categories: the subjects with WHS < = 3 (not able to perform community walking) and the subjects with WHS >3 (able to perform community walking). The Walking Handicap Scale (WHS) is an instrument that offers a quantitative method of relating the social disadvantage of walking to the impairment and disability of the patient. The metabolic test consisted of the registration of walking energy cost during a free indoor walking. For the energetic evaluation, the Body Mass Index of each subject was calculated. The energy cost of walking (CW) was measured with a portable miniature telemetry equipment (breath-by-breath-based) Oxycon Mobile (Sensormedics) [17]. Rosdahl and colleagues showed that metabolic variables, within a wide range of exercise intensities versus the Douglas bag measurements, are reliably measured with this instrument [18]. Responsiveness of Oxycon Mobile was also successfully validated in field measuring conditions, such as low temperatures, high humidity and with external wind [19]. Each test was performed in the morning, 3 hours after breakfast. The experimental procedure was the following: 5 minutes in sitting position, 6 minutes of continuous walking at a comfortable self-selected speed, and 5 minutes for recovery. All patients walked along an established route of 30 meters in length. The average speed was calculated by dividing the distance covered (m) at the time of walking. We used the term energy consumption **(**mlO_2_*Kg^−1^*Kg^1^ **)** to indicate the oxygen uptake divided by the patient’s weight. Dividing this value by the speed, we obtained the energy cost per kilogram per unit of distance covered **(**mlO_2_*Kg^−1^*min^−1^ **)**.

### Statistical Methods

To predict the walking restriction of patients in community, we performed a multivariate logistic binary regression in which the dependent variable was the WHS score (dichotomized), while the independent predictors were age, sex, kind of neurological disability, speed, distance covered, energy consumption and cost of walking. Using logistic regression models, we performed multivariate analysis aimed at identifying multiple relations between a variable of interest (walking performance of patients in community) and two or more explicative variables. Inclusion of explicative variables in the models followed stepwise procedures (forward and backward), with specific motivations for each variable. The included individual variables are reported with their Odds Ratios where appropriate and the significance of each coefficient, in the model, was examined. Non-significant variables with p-value p>0.05 were removed from the model one at a time, beginning with the variables having the highest probability levels. Every time a variable was eliminated, the integrity of the model was checked through Hosmer-Lemeshow test.

**Figure 1:**
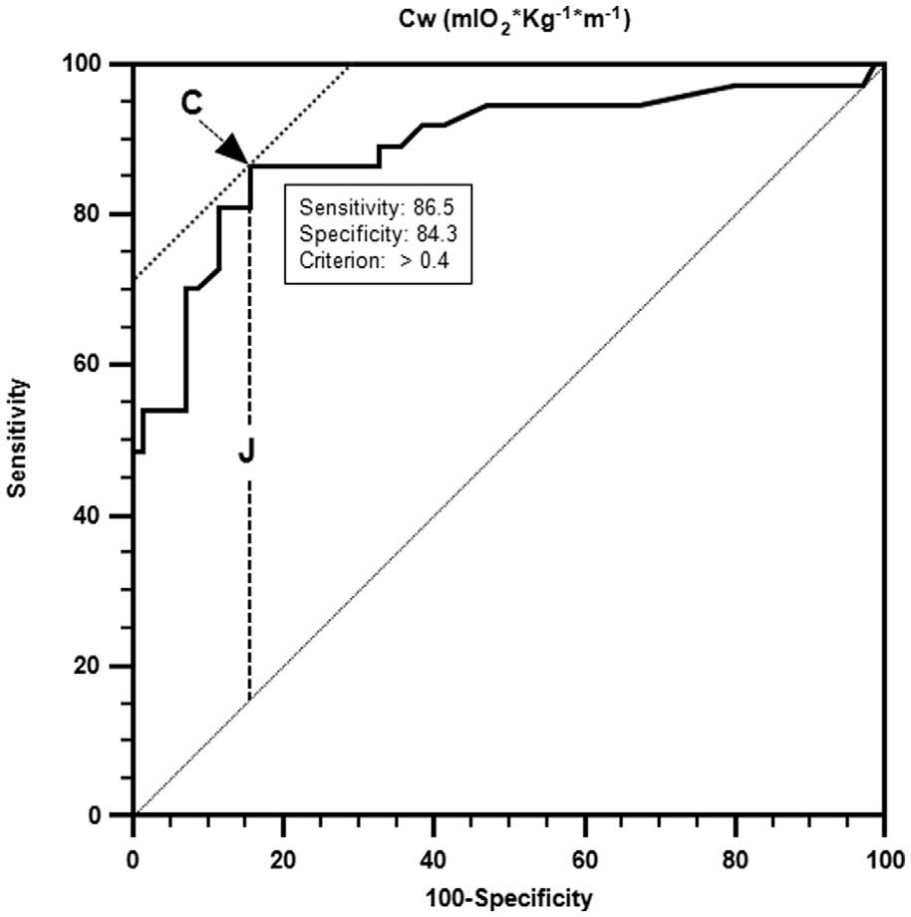
Model to identify a cut-off value of Energy Cost of walk (CW). The CW is the energy cost per kilogram per unit of distance covered **(**mlO_2_*Kg^−1^*min^−1^ **)**. C is the criterion. J = sensitivity (C)+specificity (C). J finding the best cut-off point that is equivalent to measuring the J of Youden Index. Youden Index is the greatest vertical distance between ROC curve and the diagonal line.

Once we defined a predictive model of walking in the community, we investigated whether there was a cut-off point of the energy cost of walking (the independent variables) that could predict membership of each subject in one of the two WHS categories. If a reference criterion was available, receiver-operating characteristic (ROC) analyses offered an elaborate method for the construction of cut-off points [20]. Having used a continuous variable such as Cost of Walking (CW), in which the sensitivity and specificity have for us the same statistical weight, the best cut-off point for obtaining a positive result from the test is the maximum value which can be obtained for both of these aspects of which the sum is the highest possible. This is necessary in order to identify the patients that cannot develop a walk in the context of the community. With this procedure, the determination of the cut-off point is equivalent to the achievement of the minimum value of false negative and false positive, which are dependent on mistakes in classification. The cut-off point obtained with this method has the characteristic of reaching the best expected objective, that is to say: maximize the potential for correct diagnosis and minimize the errors of classification. In the case in which c is the best cut-off point of the test results, Youden introduced the following index for ROC curve: J = sensitivity (c)+specificity (c). Moreover, finding the best cut-off point is equivalent to measuring the J of Youden Index. This index is an important synthesis of ROC curve. From the point of view of the graph, the Youden Index is the greatest vertical distance between ROC curve and the diagonal line (Fig. 1). It presents itself as having a complete and optimal potential measurement of the diagnostic capacity regarding clinical activity. ROCs describe the relation between sensitivity and specificity for different cut-off points. ROC analyses provide an evaluation of the ability of the diagnostic instruments to discriminate between health and disease.

The choice of cut-off points requires a trade-off between:

1. High sensitivity, which means the likelihood of identifying an actual risk (i.e., "Restriction in walking participation") through a positive test result.
2. High specificity, which means the likelihood of identifying a non-existent risk (i.e., "Walking independently in the community") through a negative test result.

Assuming sensitivity and specificity are of equal importance, the maximum of the Youden Index indicates an optimal cut-off point [21]. The overall ability of a measure to discriminate between healthy and diseased subjects is indicated by the magnitude of the area under the curve (AUC). We know that it exists a correlation between the positive predictive value (PPV) and the negative predictive value (NPV), and that the prevalence, which in our sample refers to people able to “walk in a social context” in any case, is unknown. It is also noted that if the prevalence of the disease in the population is high, the results of all the tests are good but, in this case, we do not know the real prevalence of the people that walk in a social context [22]–[23].

The software packages “IBM SPSS version 20” and “MedCalc version 12.1.4” were used for analyses.

## Results

**Figure 2:**
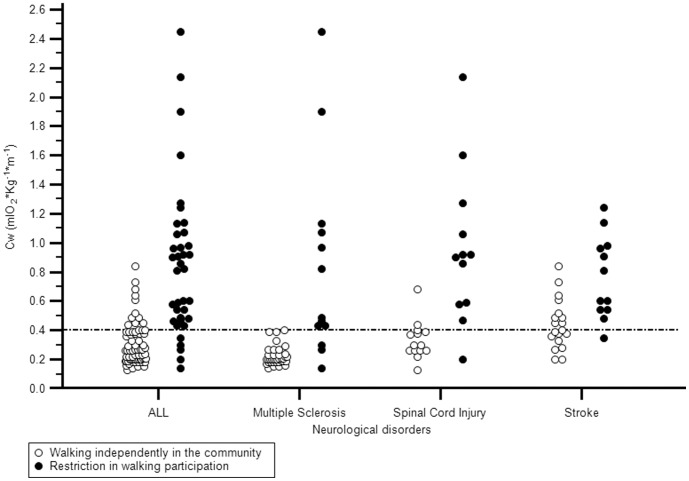
Interactive dot diagram of cut-off point of the energy cost of walking. (CW = Cost of walking).

One hundred and seven subjects were included in the study, 61 (57%) were males and 46 (43%) females: thirty-one (29%) suffering from Stroke, 26 (24.3%) from SCI and 50 (46.7%) from MS. The sample average age was 49.79±14.70 years (Stroke 62.03±11.77 years; SCI 44.92±15.56 years; MS 44.74±11.23 years), with a range of 20–84 years. Regarding clinical walking evaluation, the sample average of WHS score was 3.97±1.06 (range of 1–5), with 37 subjects whose scores were under 3, and 70 subjects whose scores were above 3 on the WHS. Walking Distance average was 203.35±129.14 meters, with a range of 12.5–528 meters; the average velocity was 33.93±21.57 m/min, with a range of 2.08–88 m/min. The mean energy consumption was 10.81±2.81 (mlO_2_*Kg^−1^*Kg^1^) and the mean value of cost of walking was.51±.515 (mlO_2_*Kg^−1^*min^−1^). For each pathology, a summary description of the collected data of the descriptive analysis of the sample and of the performance on the walking distance (WD) (m and % of predicted value), velocity (m/min), VO2 consumption, energy cost of walking (CW) with a reference to the significant range, is provided in Table 1 and Table 2. The multivariable binary logistical regression analysis has produced a statistical model with good characteristics of fit and good predictability (Table 3). The model presents a sufficient capacity of classification for each subject included in our sample (83.18% of cases). In our sample, due to the fact that both PPV and NPV are related to the sensitivity and the specificity of the test, and that they also depend on the prevalence of the disease in the population, these data, in our case, do not exist in the literature. The equation for the probability of classification model based on the measurement of energy cost (CW), which allowed us to determine the probability of "Walking Restriction in participation" for each specific value of the Energy Cost, is the following: 
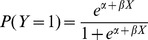
. The examples for CW = 0.39 is is the following: 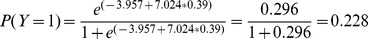. Finally, with the receiver-operating characteristic (ROC) analyses and Youden Index application, we have defined another model to identify a cut-off value of energy cost of walking that can predict the membership of each patient to one or other categories of dichotomy WHS. This model generated a cut-off value of.40 that is able to classify correctly the cases with a percentage of 85.05% (Fig. 1–2, table 4–5).

**Table 1:** Descriptive analysis of the sample.

	Gender (N)	WHS (N)						EDSS	ASIA A	ASIA B	ASIA C	ASIA D	Severe	Moderate	Mild	Timesince	Brace	Cane	Rollator
Multiple Sclerosis		1	2	3	4	5	Value										1	11	6
							**Mean**	2,79								68,02			
		0	5	8	13	24	**SD**	0,85								44,65			
	Female 33						**Mediane**	3								66			
	Male 17						**Min**	1								8			
							**Max**	4								160			
**Spinal Cord Injury**							**Value**				14	12					14	8	15
							**Mean**									35,65			
		1	3	8	4	10	**SD**									36,51			
	Female 6						**Mediane**									14,00			
	Male 20						**Min**									10,00			
							**Max**									121,00			
**Stroke**		0	2	10	7	12	**Value**						9	12	10		19	21	2
							**Mean**									21,06			
							**SD**									6,07			
	Female 7						**Mediane**									21,00			
	Male 24						**Min**									11,00			
							**Max**									33,00			

Frequency of Etiology, Gender, Walking Handicap Scale (WHS) score, Expanded Disability Status Scale (EDSS) score, International Standards for Neurological and Functional Classification of Spinal Cord Injury (ASIA) Scores, Stroke Impairment Classification (Severe, Moderate, Mild), Time since acute event (months), Walking device (Brace, Cane, Rollator).

**Table 2:** Descriptive analysis of the sample.

ETIOLOGY		Age (years)	BMI	WD (m)	WD (% predicted)	Speed (m/min)	VO_2_ (mlO_2_/min/Kg)	CW (mlO_2_*Kg^−1^*m^−1^)
**Stroke**	Mean	62.03	26.20	127.06	24.32	21.18	10.10	0.57
	Median	64.00	25.00	106.00	19.00	17.68	9.74	0.49
	SD	11.77	4.05	70.65	14.58	11.78	1.92	0.27
**Spinal Cord Injury**	Mean	44.92	25.32	148.50	24.35	24.72	10.60	0.62
	Median	46.50	25.15	138.00	19.50	23.00	9.95	0.42
	SD	15.57	3.75	79.74	17.90	13.28	3.49	0.48
**Multiple Sclerosis**	Mean	44.74	22.70	279.17	59.12	46.62	11.36	0.42
	Median	45.00	22.00	310.50	62.00	51.75	11.17	0.22
	SD	11.23	4.15	136.52	20.90	22.80	2.82	0.63
**ALL**	Mean	49.79	24.35	203.35	40.59	33.93	10.81	0.51
	Median	49.00	24.40	177.00	37.00	29.50	10.50	0.37
	SD	14.71	4.29	129.15	25.33	21.57	2.81	0.52

SD: standard deviation.

Age; Body Mass Index (BMI); Walking Distance (WD) expressed as meters and as percentage of predicted value; VO_2_ consumption (VO_2_ ) and energy cost of walking (CW = mlO2*Kg^−1^*min^−1^).

**Table 3:** Multivariable binary logistical regression analysis.

	Classification table (cut-off value p = .50)		
Actual Group (WHS dichotomized)	Predicted Group		Percent Correct
	Restriction in walking participation	Walking independently in the community	
Restriction in walking participation	24	13	64.86%
Walking independently in the community	5	65	92.86%
**Percent of cases correctly classified**			**83.18%**
	**Coefficients**		**OR (95% CI)**
**Intercept**	−3.957		
**CW** (for one point increase)	7.024*		1123.042 (68.207−18491.073)

* P<.0001.

McFadden R^2^ = .425.

Area under ROC curve = .890, 95% CI = .815−.942.

**Table 4:** Cut-off value of energy cost of walking that can predict the membership of each patient to one or other categories of dichotomy WHS.

	Classification table (cut-off value CW >.40)		
Actual Group (WHS dichotomized)	Predicted Group		Percent Correct
	Restriction in walking participation	Walking independently in the community	
Restriction in walking participation	32	5	86.49%
Walking independently in the community	11	59	84.29%
**Percent of cases correctly classified**			**85.05%**

**Table 5:** The table below summarizes the characteristics of this second model in relation to the 3 diseases.

ETIOLOGY	CW (cut-off)	SE (95% CI)	SP (95% CI)	+PV (95% CI)	−PV (95% CI)	AUC (95% CI)
**ALL**	>.40	.87 (.71–.96)	.84 (.74–.92)	.74 (.59–.87)	.92 (.83–.97)	.890 (.815–.942)
**Multiple Sclerosis**	>.40	.77 (.46–.95)	1.00 (.91–1.00)	1.00 (.69–1.00)	.93 (.80–.98)	.902 (.785–.968)
**Spinal Cord Injury**	>.44	.92 (.62–1.00)	.93 (.66–1.00)	.92 (.62–1.00)	.93 (.66–1.00)	.905 (.724–.984)
**Stroke**	>.52	.83 (.52–.98)	.79 (.54–.94)	.72 (.42–.92)	.88 (.64–.99)	.833 (.656–.942)

CI: confidence interval.

CW: energy cost of walking.

SE: sensitivity.

SP: specificity.

+PV: positive predictive value.

−PV: negative predictive value.

AUC: area under the ROC curve (maximum = 1.0).

## Discussion

The absence of definitive evidence to support the choice of the best clinical test or tests, which may be used in the examination of neurological patient to determine the ability of walking in the community and “the walking restriction in participation”, remains a matter of clinical judgement.

The literature on physical rehabilitation refers, frequently, to patient’s motivation in explaining differences in outcome among patient groups with similar pathologies. Participation may involve returning to previous activities and groups which were, and still are, an important target to the stroke survivor. The goal of community re-integration or community participation is to facilitate the transformation of ‘stroke survivors’ to ‘stroke thrivers’. Community reintegration requires an environment that empowers stroke survivors and their family/caregivers to develop personal goals and the methods to achieve them. After discharge from rehabilitation wards, it may be important to make a correlation within objective parameters of walking, the assessment of impairments and the outcome of activity and participation of individuals, and the influence on health-related quality of life. The measurement of therapeutic outcome, in relation to the social advantage for the patient, would allow more efficient standardization of treatment and services but many environmental dimensions influence the walking performance in the community [24]–[27]. Our result could help to find out if the value of the energy cost of walking, in a mixed group of neurological patients, could predict and correlate with the walking performance in the community expressed by WHS; furthermore, these data could be used to predict the patients' outcome. The metabolic cost of walking seems to be one of the main factors that bind the stride characteristics of the individual patient with the possible performance of the road under real conditions. For this reason, reduced cardio respiratory fitness may be a secondary factor that limits the transfer of walking skills obtained during rehabilitation back into the community environment [28]. The results of our research reveal that, for our subjects, the CW could be a good predictor of walking performance in the community, compared with the score of WHS. We have been also identifying a cut-off value of CW cost, which distinguishes between those who can walk in the community and those who cannot do it. The discriminative ability of a test, i.e. its ability to separate properly the study population into "sick" and "healthy", is proportionate to the extent of the area under the curve (ROC Area Under Curve, AUC) and is equivalent to the probability that the result of a test of an individual, chosen at random from the group of patients, is higher than the one chosen at random from the group of non-sufferers [21], [22]. The evaluation of a test is carried out through the AUC, which gives equal importance to the sensitivity and specificity (as in our work) while, in many cases, it is necessary to differentiate the weight to be assigned to these parameters. A more suitable approach may be adopted by taking into account the relation between sensitivity and specificity, that is studying the ROC curve. The use of ROC curve represents a more "flexible" criterion as it offers the ability to view, given a choice of Specificity value, the corresponding sensitivity value and vice versa [23]. In our work, the multivariate binary logistic demonstrated the association between energy cost with the result of dichotomized WHS-score analysis. It seems that this parameter summarizes the variables of diagnosis of disease, age and sex. It is known that, in normal gait, velocity is the most important factor in determining oxygen uptake of walking and is independent of age or sex [29], [30]. In addition, the literature shows that the energy cost of walking is highly related to factors that alter the biomechanics of the hand, such as paralysis, spasticity, and muscle co-contractions of the aetiology of the disease [31]–[33]: an indirect evidence is that the orthotic and therapeutic solutions, which try to reduce the energy expenditure of walking impairment, result from the type of pitch and biomechanical characteristics of the space-step and not from the diagnosis [34]–[37]. Our results of the CW in MS and SCI subjects are homogeneous but, in stroke people, the value of CW increased slightly, and this aspect could be justified with the significantly difference of ages between the groups. In post stroke patients, the elderly could reduce the motivation to have a correct participation in the community. This cut-off could be useful when discharging patients from the rehabilitation setting to define better the prognosis regarding the participation in the community and to help their families with a correct information regarding the choice of aids and home modifications. Furthermore, the result could be useful during the post-hospital rehabilitation treatment in order to intensify the work to improve walking efficiency, thus improving the performance capacity. Many people who suffered with a stroke have a low level of satisfaction in participation, after they have been discharged from the hospital and have returned to the community [38]. As many as 39% to 65% of community-dwelling people with stroke report limitations in activities and restrictions in community participation. In one study, Nancy Mayo and colleagues found out that (70%) had a restriction in travelling within and beyond the community. Also, 72% of the stroke people lacked an important and meaningful activity to fill the day, whether it be social, recreational, or occupational [39]. A first limitation of this study is secondary to single-centre that enrolled patients for this study. The second limitation could be related to the lacks of cross languages translation and validation of WHS for SCI and MS. In particular, the assessment of the CW evaluation could need an active help and motivation by the subject that performs it. The result of this study will enable the design of new observational longitudinal research through which it could be verified, on a large scale, whether these mathematical models are supported by clinical data with a long time follow-up.

### Conclusion

Several Authors have shown the efficacy of gait training on improving walking function in a variety of neurological diagnoses, but the process aimed at restoring walking function is challenged by the complexity and variability inherent in these disor­ders. Many factors could interfere with walking recovery in neurological diseases. Our results reveal that the capacity and/or the inefficiency CW could interfere with walking independently in the community, furthermore, in our subjects, we have been also identifying a cut-off value of CW. These values could be used to predict the ability to walk in the community when discharged from the rehabilitation units, and to adjust the rehabilitative treatment to improve the performance. In order to confirm the present statistical approach, further multicentre clinical trials should be conducted, in the future, involving a high number of people, with a long-term follow-up.
